# Acute Stent Thrombosis Associated with Eptifibatide-Induced Profound Thrombocytopenia

**DOI:** 10.1155/2020/8386709

**Published:** 2020-06-09

**Authors:** Amer Aljundi, Alaa Rahhal, Wafer Dabdoob

**Affiliations:** ^1^Pharmacy Department, Heart Hospital, Hamad Medical Corporation, Doha, Qatar; ^2^Cardiology and Cardiovascular Surgery Department, Heart Hospital, Hamad Medical Corporation, Doha, Qatar

## Abstract

**Background:**

Eptifibatide is an inhibitor of the platelet glycoprotein (GP) IIb/IIIa receptor that is commonly used in patients undergoing percutaneous coronary intervention (PCI).

**Case:**

We describe a case of a 62-year-old female patient admitted with acute ST-elevation myocardial infarction (STEMI) treated by primary coronary intervention (primary PCI) with a drug-eluting stent placement. She developed profound thrombocytopenia within 8 hours of first administration of eptifibatide and subsequent acute stent thrombosis next day. Other causes of thrombocytopenia were excluded and intravascular ultrasound (IVUS) showed good stent expansion and opposition to the coronary wall. Platelet count gradually returned to normal after discontinuation of eptifibatide.

**Conclusion:**

Although Eptifibatide has been associated with the development of thrombocytopenia, to the best of our knowledge, this is the first case report in the medical literature that associates acute stent thrombosis and eptifibatide-induced thrombocytopenia.

## 1. Introduction

There are several agents that are used in the treatment of acute coronary syndromes (ACS) that were reported to cause drug-induced thrombocytopenia [1, 2]. Eptifibatide is a synthetic cyclic heptapeptide and a selective inhibitor of the platelet glycoprotein (GP) IIb/IIIa receptor that blocks the final common pathway of platelet aggregation. It is commonly used in patients with acute coronary syndromes and in those undergoing PCI to reduce the risk of acute cardiac ischemic events [3–6].

Recent literature has suggested an association between eptifibatide exposure and the development of thrombocytopenia. However, to the best of our knowledge, this is the first case report in the medical literature that associates acute stent thrombosis and eptifibatide-induced thrombocytopenia.

## 2. Case Presentation

A 62-year-old female with a history of diabetes mellitus and hypertension presented to the emergency department with a two-hour history of retrosternal chest pain radiating to both shoulders and associated with profuse sweating and vomiting. She denied any previous history of blood dyscrasia or thrombocytopenia. She had no history of drug abuse and denied any history of a previous hospitalization where she may have received heparin or eptifibatide. She does not have any known allergies. Her past medication history included the use of amlodipine 5 mg daily, atorvastatin 20 mg daily, metformin 500 mg daily, aspirin 100 mg daily, carvedilol 25 mg twice daily, and lisinopril 20 mg/hydrochlorothiazide 12.5 mg daily.

Vital signs at presentation included a temperature of 36.8°C, regular pulse of 98 bpm, brachial blood pressure of 140/70 mmHg, respirations of 20 per minute, and oxygen saturation of 98% on room air. The physical exam demonstrated an alert and oriented patient in moderate distress from chest pain. There were no signs of peripheral edema or cyanosis. The patient had bilateral basilar crackles at the bases. The heart was regular, with no murmurs, rubs, and gallops. The abdomen was soft with no organomegaly.

**Figure 1:**
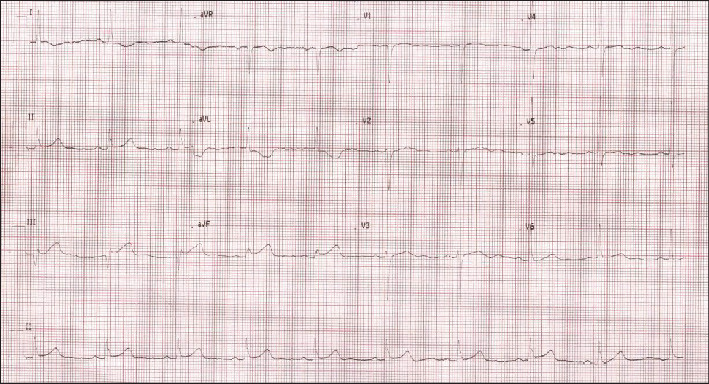
Electrocardiogram (ECG) showing ST-segment elevation in lead II, III, aVF, Q wave in III, and reciprocal ST-segment depression in I and aVL at presentation.

Her electrocardiogram (ECG) showed ST-segment elevation in lead II, III, aVF, Q wave in III, and reciprocal ST-segment depression in I and aVL (Figure 1). Transthoracic echocardiography showed inferior left ventricular (LV) wall motion akinesia with normal LV systolic function (as demonstrated by an LV ejection fraction of 55-60%) and mild concentric LV hypertrophy. Other findings included a mild mitral regurgitation with normal other valves and chambers. At baseline, the patient had a white blood cell count of 12.000/mm3, a hemoglobin level of 13.9 g/dL, and a platelet count of 378,000/mm3. Cardiac markers were creatine kinase myoglobin (CK MB) level of 87.40 ng/ml and troponin T level of 4040 ng/mL. The values of prothrombin time (PT) and activated partial thromboplastin time (aPTT) were within normal limits. Similarly, liver function tests and kidney function tests were within normal limits. Due to the patient's ischemic symptoms and ECG changes consistent with an acute inferior STEMI, she was taken for urgent cardiac catheterization. Coronary angiography revealed a dominant RCA with a 99% stenosis with a thrombolysis in myocardial infarction (TIMI) grade 0 flow and a 40% stenosis of the left circumflex artery (LCx). Angiography also showed normal left main coronary artery (LMCA) and left anterior descending (LAD) coronary artery.

**Figure 2:**
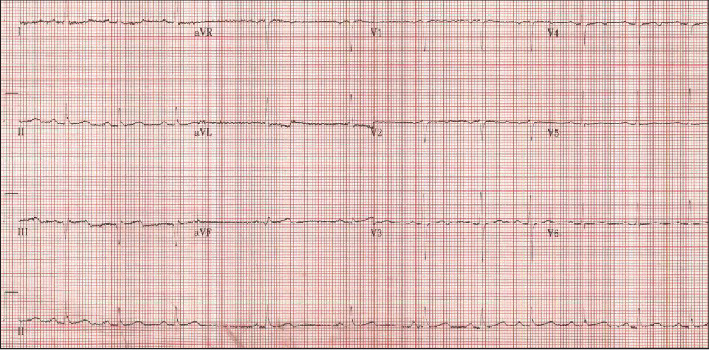
ECG showing resolution of ST-segment elevation after PCI to RCA.

Following our local protocol, before catheterization, the patient received aspirin 300 mg; clopidogrel 600 mg; one dose of intravenous unfractionated heparin 7000 units; and eptifibatide 180 *μ*g/kg as a bolus dose then 2-*μ*g/kg/min infusion. A 3.0 × 24 mm XIENCE™ Everolimus Eluting Coronary stent was deployed in the distal RCA with a very satisfying angiographic result (TIMI grade 3 flow) with no complications. Once flow was restored in the RCA, the patient became pain-free with the resolution of her ST-segment elevation (Figure 2). She was then transferred in a stable condition to the coronary intensive care unit (CICU). Postpercutaneous coronary intervention (PCI) medications included dual antiplatelet therapy (DAPT) with aspirin 100 mg daily and clopidogrel 75 mg daily, ramipril 5 mg daily, metoprolol tartrate 25 mg twice daily, rosuvastatin 20 mg daily, and the eptifibatide intravenous infusion was to be continued for 18 hours.

**Figure 3:**
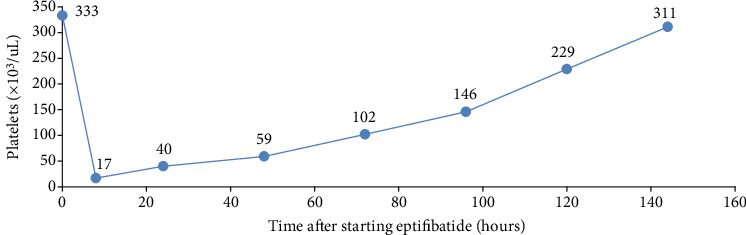
Platelet count during hospital stay.

Approximately eight hours post-PCI and eptifibatide initiation, the patient developed a profound thrombocytopenia, with her platelet count dropping by over 90% from baseline to 17,000/mm3. The rest of her complete blood count at this time included hemoglobin 13.2 g/dL, hematocrit 39.6%, and white blood cell count 18.8 × 109/L. The platelet count trend through hospitalization is summarized in Figure 3.

After the acute platelet drop, eptifibatide was discontinued. Intravenous lepirudin 14.5 mg/h was initiated due to a high clinical suspicion for heparin-induced thrombocytopenia (HIT).

**Figure 4:**
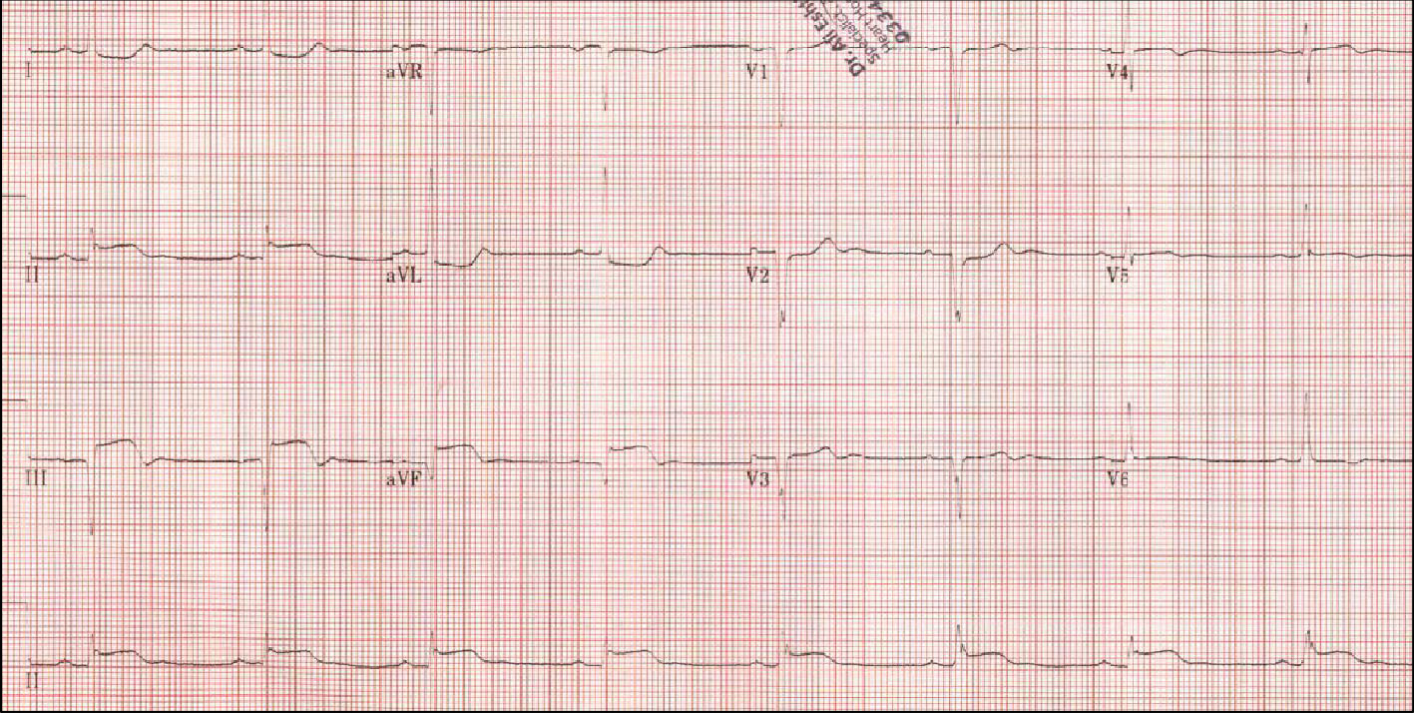
ECG showing diffuse ST elevation in lead II, III, aVF, and reciprocal ST-segment depression in I and aVL.

**Figure 5:**
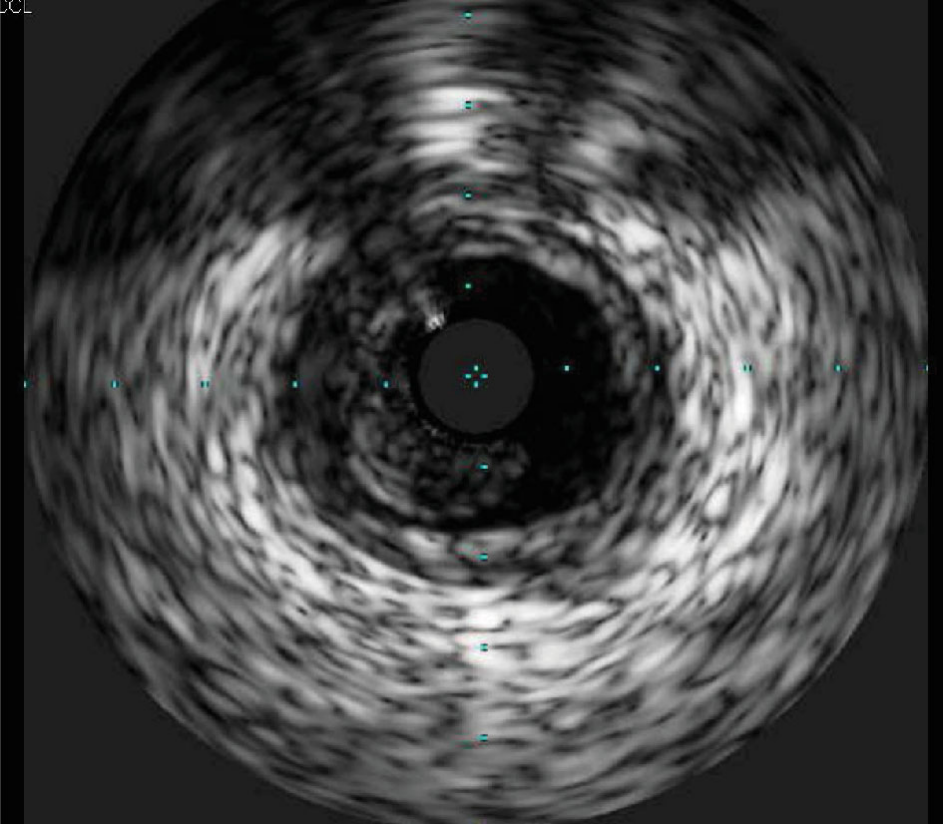
IVUS showing thrombus in mid RCA at 5 to 11 o'clock.

**Figure 6:**
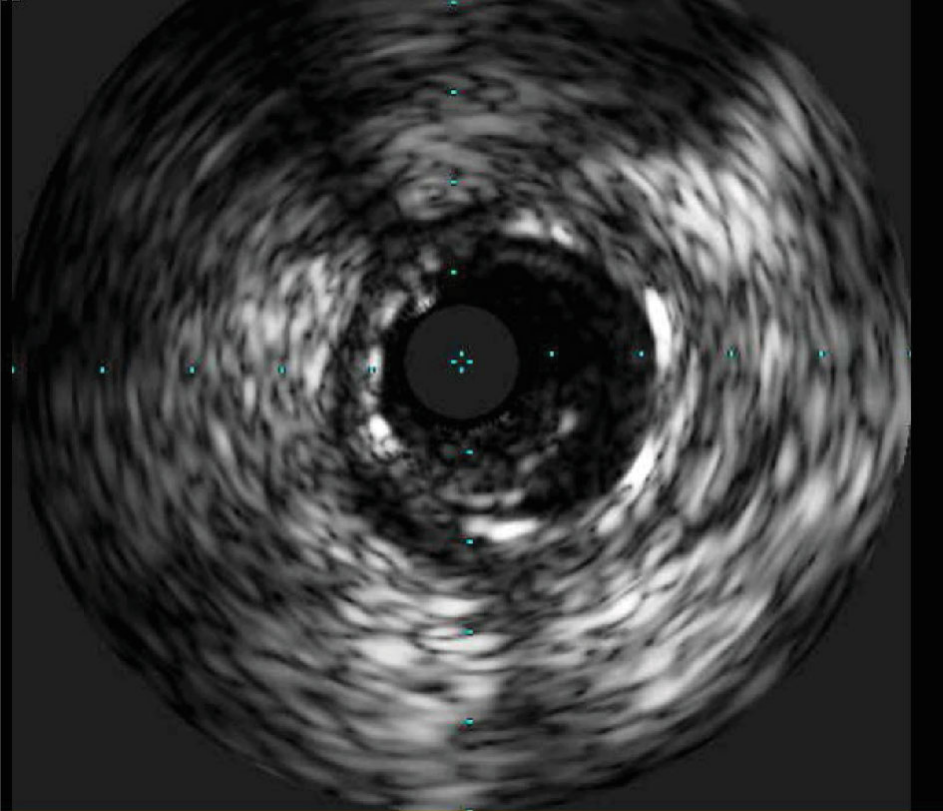
IVUS showing thrombus at 3 to 8 o'clock within the stent.

**Figure 7:**
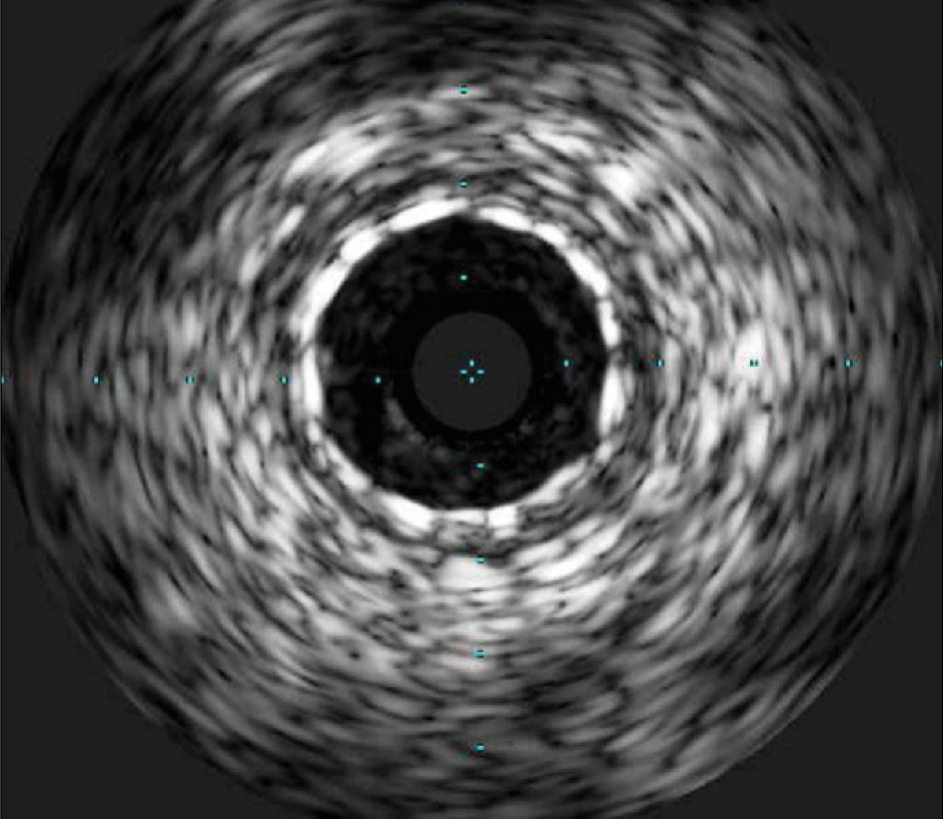
IVUS showing well-expanded and opposed stent.

On the next day (approximately 18 hours after primary PCI), our patient developed chest pain with nausea and vomiting. Her electrocardiogram (ECG) showed diffuse STEMI of the II, III, aVF, and reciprocal ST-segment depression in I and aVL (Figure 4). Urgent angiography revealed thrombosed mid and distal RCA with intravascular ultrasound (IVUS) study conforming thrombus formation (Figures 5 and 6). Export catheter™ was utilized to extract clot fragments with satisfying results (TIMI grade 2 flow). The subsequent IVUS study confirmed excellent stent expansion and apposition to the vessel indicating the optimal stent deployment (Figure 7).

## 3. Discussion

Eptifibatide is a potent adjunct medication that significantly improves outcomes in patients undergoing PCI among those presenting with ACS [3–6]. Eptifibatide works by preventing the binding of fibrinogen, von Willebrand factor, and other ligands to the GP IIb/IIIa receptor, thereby reversibly inhibiting platelet aggregation and subsequent thrombosis [1, 2]. Drug-induced acute profound thrombocytopenia can be defined as a decrease in platelet count to below 20,000/mm3 within 24 hours of exposure to the drug, which was the case in our patient having reached a low as 17,000/mm3 [7].

Our patient was exposed to several agents other than eptifibatide that are well known to be associated independently with profound thrombocytopenia, including heparin and clopidogrel [8]. These agents are usually coadministered before PCI which can make it difficult to discern causation in the setting of drug-induced thrombocytopenia.

Treatment with GP IIb/IIIa antagonists has been reported to be associated with pseudothrombocytopenia [9]. Pseudothrombocytopenia is an artificial laboratory process resulting from the adherence of platelets to leukocytes. This process typically occurs when blood samples are collected in EDTA tubes. Pseudothrombocytopenia in our scenario was ruled out by confirmation of thrombocytopenia in an EDTA-free tube and by direct review of the peripheral blood smear which showed no signs of platelet clumping. Moreover, there was no evidence of microangiopathic hemolytic anemia consistent with thrombotic thrombocytopenic purpura–hemolytic uremic syndrome (TTP-HUS) due to the administration of clopidogrel [10]. Additionally, other than thrombocytopenia, our patient did not manifest any other hallmarks of the disease which include fever, neurologic manifestations, and renal insufficiency. Moreover, our patient received only one loading dose of clopidogrel 600 mg six hours before the drop in her platelet count making it unlikely that clopidogrel was the culprit for the observed thrombocytopenia.

Use of the Naranjo scale to assess the probability of drug adverse events also indicated a probable relationship between the administration of eptifibatide and the development of profound thrombocytopenia (score of 5), while the relation was possible with heparin and clopidogrel (score 1) [11].

Heparin is well known to be associated with thrombocytopenia; however, the timing of thrombocytopenia in this patient was consistent with eptifibatide-induced thrombocytopenia as it occurred within less than 24 hours of stating eptifibatide. Heparin-induced thrombocytopenia (HIT) type I usually develops early within the first two days of starting heparin and is usually mild and transient (rarely <100,000 platelet/mm3). Heparin-induced thrombocytopenia type II, on the other hand, is an immune-mediated reaction with a typical onset of 5-10 days after the administration of heparin without previous heparin exposure and can be as early as hours due to circulating heparin/platelet factor 4 from a previous exposure [12]. Our patient had a severe form of thrombocytopenia ruling out HIT type I, while the time frame for developing thrombocytopenia was not consistent with HIT type II as our patient had no previous exposure to heparin. Additionally, ELISA immunoassay was performed and was negative for heparin-platelet factor 4 antibodies. The patient's platelet count started to improve after discontinuing eptifibatide. These factors suggested that eptifibatide was the most likely cause of thrombocytopenia associated with acute stent thrombosis.

The mechanisms by which GP IIb-IIIa inhibitors cause thrombocytopenia are poorly understood. One possible mechanism is the formation of antibodies to a group of epitopes, called ligand-induced binding sites (LIBSs), which are normally hidden on the surface of the platelet. Binding of the GP IIb-IIIa inhibitor to the receptor induces a conformational change exposing LIBSs in the platelet. This permits the binding of the anti-LIBS antibodies and can eventually lead to subsequent clearance of platelets by the reticuloendothelial system [13].

A proposed mechanism for thrombosis, however, is that LIBS antibodies can activate platelets by stabilizing the open, or active, conformation of the GP IIb/IIIa receptor, enabling the binding of multivalent ligands such as fibrinogen. Antibody-mediated fibrinogen binding can lead to bridging of adjacent platelets and platelet activation responses with subsequent generation of cell-surface procoagulant activity [14].

## 4. Conclusion

Eptifibatide is known to cause severe thrombocytopenia; however, this the first report of this being associated with subsequent acute stent thrombosis. Despite being uncommon, it is a clinically important complication of eptifibatide. This report highlights the importance of close monitoring platelet counts, before and after starting eptifibatide. A high index of suspicion of acute stent thrombosis should be raised if a patient develops new onset chest pain following successful PCI in the setting of eptifibatide-induced thrombocytopenia.

## Data Availability

The data used to support the findings of this study are included within the article.
